# An alternative protocol for *Plasmodium falciparum* culture synchronization and a new method for synchrony confirmation

**DOI:** 10.1186/1475-2875-12-386

**Published:** 2013-11-01

**Authors:** Ronnie A Childs, Jun Miao, Channe Gowda, Liwang Cui

**Affiliations:** 1 Department of Entomology, Pennsylvania State University, University Park, PA 16802, USA; 2 Department of Biochemistry and Molecular Biology, Pennsylvania State University, College of Medicine, Hershey, PA 17033, USA

**Keywords:** SyBr Green I, *Plasmodium falciparum*, Egress, Invasion, Culture synchronization

## Abstract

**Background:**

Although the Percoll/sorbitol synchronization method is widely accepted, its use for achieving tight synchronizations is cumbersome. In addition, subsequent conclusions on the synchrony status are often based on visual inspection of parasites and few reports provide an unbiased estimate confirming the degree of synchrony. This report presents a simpler synchronization procedure and offers an objective method to validate parasite synchrony.

**Methods:**

Parasite synchronization was performed by culturing late-stage schizont parasites for a defined period of time, subjecting them to Percoll density centrifugations, and collecting the newly formed rings. Repeating the process several times on the un-egressed schizonts maximizes the recovery of several synchronized ring-stage parasite populations. The culture synchrony for each population was verified by allowing the synchronized rings to mature to late-stage schizonts and collecting ring-stage sample aliquots at three-hour intervals for nine hours. The aliquots were then measured, using the SyBr Green I assay, to determine when the ring-stage parasitaemia stops increasing.

**Results:**

Quantitative measurements of ring-stage parasites showed that under the conditions described, a four to six-hour synchrony period is obtained.

**Conclusion:**

By taking advantage of *Plasmodium*’s periodic lifecycle in erythrocytes, it is shown that Percoll density centrifugation alone is sufficient to tightly synchronize cultures with minimal parasite loss. In addition, the degree of culture synchrony is validated using the SyBr Green I assay.

## Background

The malaria parasite possesses a complex life cycle that can be separated into two main phases, the mosquito phase and the mammalian phase. Under appropriate conditions, the parasite cycles in the human erythrocyte from merozoite stage (MS) to ring stage (RS), trophozoite stage (TS), and schizont stage (SS) [1,2]. Failure of schizonts to egress from the erythrocytes or invade new ones, leads to parasite death [3]. Although parasite egress and invasion are crucial for the parasite’s survival, the underlying mechanisms are not fully understood. Investigation of these events usually requires that the parasites within the culture are at equivalent developmental stages or “synchronized” [4]. The degree of synchrony required can vary depending on the target under investigation and goals of the study.

One of the most common procedures used to obtain synchronized cultures is Percoll density centrifugation. The original isopycnic Percoll method, established in 1981 for *Plasmodium berghei*[5] and 1983 for *Plasmodium falciparum*[6], was unable to tightly synchronize parasite stages because it could not separate late trophozoites and early schizonts from late schizonts. The sorbitol synchronization method, developed in 1979 [7], relied on lysing trophozoites and schizonts while allowing for continued growth of the RS. Several rounds of sorbitol treatment were necessary and, even then, a tight synchronization was difficult to achieve by this procedure. To overcome these limitations, a combination of two methods was established in 1985 by Kutner *et al*. [8]: the isolation of matured parasites by centrifugation on Percoll cushions, and sorbitol treatment of schizont stages. They reasoned that sorbitol altered the buoyant density of mature stages due to increased expression of permeability pathways, allowing a higher degree of separation in a Percoll gradient. Recently, Radfar *et al.* modified this protocol by alternating the Percoll and sorbitol treatments during specific stages to achieve tight synchronization at high parasitaemia [9].

The repeated Percoll/sorbitol treatments needed to synchronize a culture, as well as the difficulty in achieving tight synchronization following such treatments, prompted a search for a method that did not poison the culture, reasonably defined the zero hour start point for RS, tightly synchronized the culture, and was easy and reproducible. Here, an alternative Percoll synchronization protocol is reported and evidence is provided showing that the use of the SyBr Green I assay is valid for measuring RS parasites and culture synchrony.

## Methods

### Parasite culture and preparations

*Plasmodium falciparum* 3D7 parasites were cultured in a complete medium at 1% haematocrit at 37°C in a 5% CO_2_/3% O_2_/balanced N_2_ gas mixture as described previously [10]. Human red blood cells (RBCs) were washed three times, stored at 4-8°C, and used within three weeks. The haematocrit was measured from a packed RBC volume that was centrifuged in a swinging bucket rotor at 2,000x g for 5-min at room temperature. Schizonts were isolated on Percoll cushions centrifuged at room temperature in a swinging bucket rotor at 2,000x g for 5-min. For RS parasite analysis, aliquots were layered on top of 70% Percoll in 1.6 ml tubes and centrifuged at 4,000x g for 5-min. Pellets were washed once with RPMI 1640 medium and immediately frozen at −20°C until used.

### Synchronization of parasite culture

For culture synchronization, parasites were cultured to at least 10% parasitaemia in T-75 flasks containing 50 ml medium at 1% haematocrit. When most of the parasites matured to SS, the culture was loaded on a 20% over 60% Percoll cushion and centrifuged for 5 min at 2,000x g. Schizonts from the top of 60% Percoll was collected (without washing) and re-suspended in complete medium containing 2% RBCs at a final Percoll concentration of less than 6%. This culture was incubated at 37°C for 3-hr in a 5% CO_2_/3% O_2_/balanced N_2_ gas mixture, then separated on a 20% over 70% Percoll cushion. The pellet contained newly infected RBCs or rings (S1), while the layer on top of 70% Percoll contained schizonts. The schizonts were returned to culture as above and the process was repeated two additional times, thereby producing additional batches of highly synchronized RS cultures (herein called S2 and S3).

To determine the degree of synchrony for these parasite batches (S1, S2, or S3) or for use in egress and invasion experiments, the synchronized RS cultures must develop into schizonts. Therefore each synchronized RS pellet isolated above was re-suspended in 20 ml complete medium and the time was documented as start time equals 0-hr. The RS sample was then incubated for 8-hr as above, pelleted by overlaying onto a 40% over 70% Percoll cushion, and washed with RPMI 1640. This important Percoll step removes trophozoites that may have escaped the initial separation process. To ensure that the high parasitaemia culture is not depleted of nutrients, pellets were re-suspended in 50 ml of complete medium, incubated for 20-hr, then aspirated (not pelleted) and replaced with 50 ml of fresh complete medium. The incubation continued such that the total incubation time from the above-mentioned documentation time was 48-hr (i e, another 20-hr). At harvest the synchronized schizonts were re-isolated using a 20% over 70% Percoll cushion and used immediately. Please see Additional file 1 for the detailed procedure.

A standard Percoll/sorbitol synchronization method was also used for comparison. Briefly, schizonts were isolated over a 40% over 60% Percoll cushion and allowed to infect RBCs for a period of 6- to 12- hr [10]. The resulting RS were subjected to 5% sorbitol treatment for 5- min at 37 °C. This process was repeated two to three times. Following the final sorbitol treatment of schizonts, the culture was washed and re-suspended in complete medium containing 1% RBC. Parasites not treated with sorbitol were also cultured as a control for comparison (herein described as mock culture or MS1).

### Confirmation of culture synchrony

To determine the degree of culture synchrony, schizonts were isolated from a 20% over 60% Percoll cushion, re-suspended in at least 20 volumes of complete medium at 1% haematocrit, then incubated at 37°C. One ml aliquots were removed at 0-, 3-, 6-, 9-hr and each was over-layed onto a 70% Percoll cushion (0.5 ml) and centrifuged to recover RS parasites in the pellets. The pellets were washed with RPMI 1640 and frozen for storage. They were later thawed and processed for SyBr Green I analysis. Please see Additional file 2 for the detailed procedure.

### SyBr Green assay

The SyBr Green I assay was carried out as previously described with the following modifications [11-17]: all parasite pellets obtained from 0.5 ml culture aliquots were thawed in 0.5 ml re-suspension buffer (25 mM Tris, pH 7.5, 10 mM ETDA, 20 μg/ml RNase A), making the final haematocrit of 1%. Aliquots of 100 μl were mixed with 100 μl of ice cold SyBr Green I solution (25 mM Tris, pH 7.5, 10 mM EDTA, 0.01% saponin, 0.1% Triton X-100, 0.2 μl/ml SYBR Green I) in a 96-well black plate (Corning #3916). The plate was incubated at room temperature for 1-hr on a rotating platform. The relative fluorescent intensity (RFI) readings were measured (excitation 485 nm/emission 520 nm) using a Fluostar Optima Microplate Reader (BMG Labtech).

## Results

### Parasite synchronization by Percoll density centrifugation

The synchronization method described here takes advantage of Percoll’s ability to fractionate the RS from matured parasites. When parasite cultures are centrifuged on 60% Percoll cushions, the SS population is retained on the top layer and used to initiate new infections. Purified SS vary in maturity, and the time of merozoite egress from infected RBCs (iRBCs) could last up to 9-hr following the initial Percoll isolation. Thus, by repeating the above process additional times at defined intervals, most of the released merozoites are captured as synchronized rings in subsequent isolations.

The time window for the incubation can range from 1-hr to several hours, depending on the desired degree of synchrony. A 3-hr incubation time interval was used on schizonts to illustrate this principle (Additional file 3). For cultures used in these experiments, the second fraction (3- to 6- hr) had the highest number of rings and resulted in the highest parasitemia (Additional file 4). Parasites isolated as outlined above maintained a highly synchronous 48-hr developmental cycle and began the egress/invasion process at 48-hr from the time they were harvested (Additional file 5).

In addition to the ease of application, another attractive advantage of this method is that the initial capture of schizonts (20% over 60% Percoll cushions) needs only to occur at an approximate time to the onset of egress. For example, if the initial schizont capture is too early (i e, mid-stage schizonts rather than late-stage schizonts), a S1 population of rings (0- to 3- hr) might not be recovered within the 3-hr egress/invasion period. Alternatively the recovered parasitaemia may be too low for use in experiments. However, subsequent schizont isolations will produce synchronized rings populations (i e, S2 at 3- to 6- hr, and S3 at 6- to 9- hr) each having a defined 0-hr start time and at higher parasitaemia.

### Confirmation of culture synchronization

**Figure 1:**
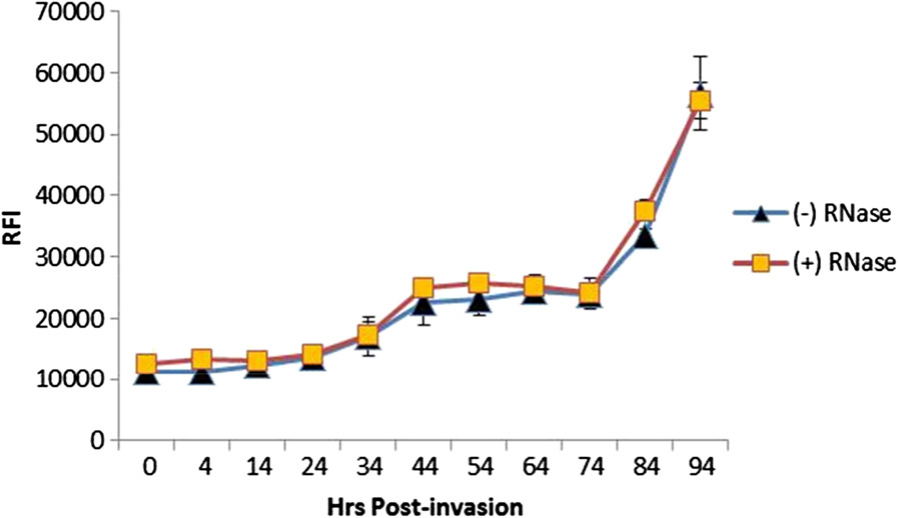
**SyBr Green analysis of parasite lifecycle.** The culture was initiated by incubating mature schizonts with RBCs in a complete medium at 1% haematocrit. Aliquots were taken at the indicated time points (0- to 94-hr) and centrifuged on 40% Percoll cushions to remove haemozoin and cell debris. Cell pellets were then used in the SyBr Green I assay either with or without RNase A treatment to determine the effects of RNA transcripts on RFI readings. Three biological replicates were run in triplicate and averaged, and the standard deviations calculated. Please see Additional file 5 for photos of lifecycle stages.

To determine the degree of synchrony, a SyBr Green based assay was adopted. First, periods within the parasite’s lifecycle when the relative fluorescent intensity (RFI) values did not increase during the culture proceedings were identified. This allowed for the determination of which parasite stages could be quantified using the SyBr Green assay. Earlier studies showed that parasite DNA replication does not occur until early TS, (i e, ~28-hr after parasite invasion) [18]. Measurments from SyBr Green I fluorescence showed that RFI did not increase until at least 24-hr after invasion, which corresponds to RS (Figure 1 and Additional file 6). The lack of an RFI increase during ring development justifies the use of the SyBr Green I for both detection and quantification of RS parasites. Incubation of the lysates with RNase A showed that RNA did not have any noticeable effect on the RFI values.

**Figure 2:**
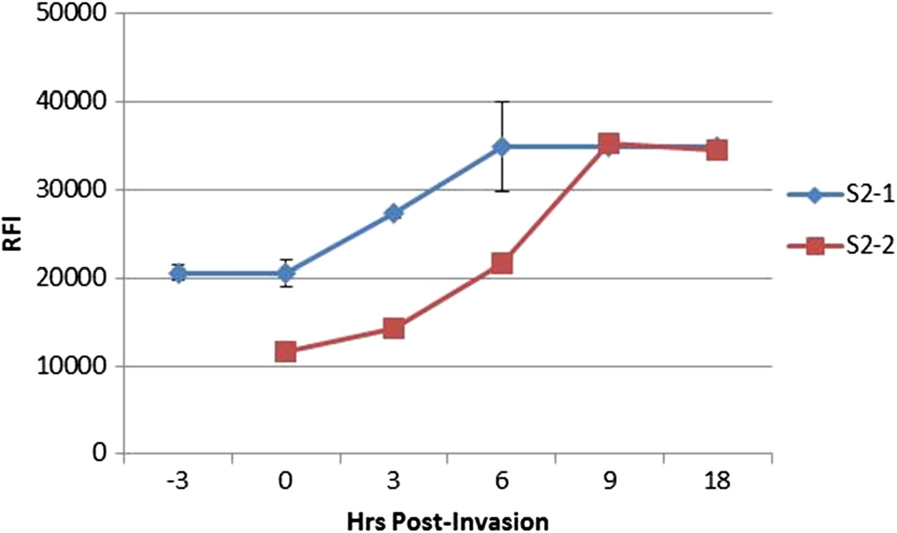
**Synchrony confirmation of samples from Percoll synchronization method.** The synchrony of two S2 aliquots (3- to 6-hr, see Additional file 1) was confirmed by incubating the RS samples in complete media until they reached SS. The 0-hr denotes the calculated start of egress/invasion. The matured SS parasites were isolated over a 20%/60% Percoll cushion at the calculated egress time (S2-2) or 3-hr before the calculated egress time (S2-1) and re-suspended in complete media containing 1% RBC. Aliquots were taken (0.5 ml) at the indicated time points and ran over a 70% Percoll cushion, washed, and frozen. Samples were later re-suspended to a 1% haematocrit using resuspension buffer (0.5 ml) and 0.1 ml aliquots were assayed in triplicate using the SyBr Green assay format.

Next, to verify culture synchrony, late SS from synchronized cultures were purified and incubated with fresh RBCs. Aliquots were removed every 3-hr and centrifuged over a 70% Percoll cushion. The resulting pellets, which contained newly formed RS parasites, were used in the SyBr Green I assay. The RFI increase of the pelleted aliquots correlates with an increase in newly formed RS, indicating egress and invasion has occurred. Likewise, once egress and invasion ceases the RFI values will stop increasing because new rings are not formed. The period of time it takes for the RFI values to stop increasing determines the degree of culture synchrony. Figure 2 confirms the synchrony of a culture synchronized using the method described here. Photos for the synchronization and confirmation protocols are attached in Additional files 7, 8, 9, 10, 11, and 12.

Aliquots from preparation S2-2 (Figure 2) were taken at the start of the estimated egress time. Aliquots from preparation S2-1 were taken 3-hr before the onset of egress. Notice the delay in the onset of egress when aliquots were removed 3-hr before the start of the egress period. Rings are not formed since egress has not occurred, thus no RFI increase is observed. The delay therefore indicates that the detection method is functioning properly. Figure 2 illustrates how the SyBr Green I method can determine the synchrony of two cultures. Both cultures (S2-1 and S2-2 preparations) are synchronized within a range of 4- to 6-hr. There was no significant increase in RFI readings prior to 0-hr and after 6-hr (S2-1) or before 3-hr and after 9-hr (S2-2). Although preparation S2-2 shows a slight RFI increase during the 0- to 3-hr period, this increase was compared to the average increase from 0- to 9-hr using a one tailed *t*-test, assuming unequal variances. The average RFI increase from 0- to 3-hr was negligible compared to the RFI increase from 0- to 9-hr (*P* = 0.0002), indicating a 4- to 6-hr synchrony period. This procedure can be used to verify the synchrony of any culture regardless of synchronization method.

### Comparing synchronization procedures

**Figure 3:**
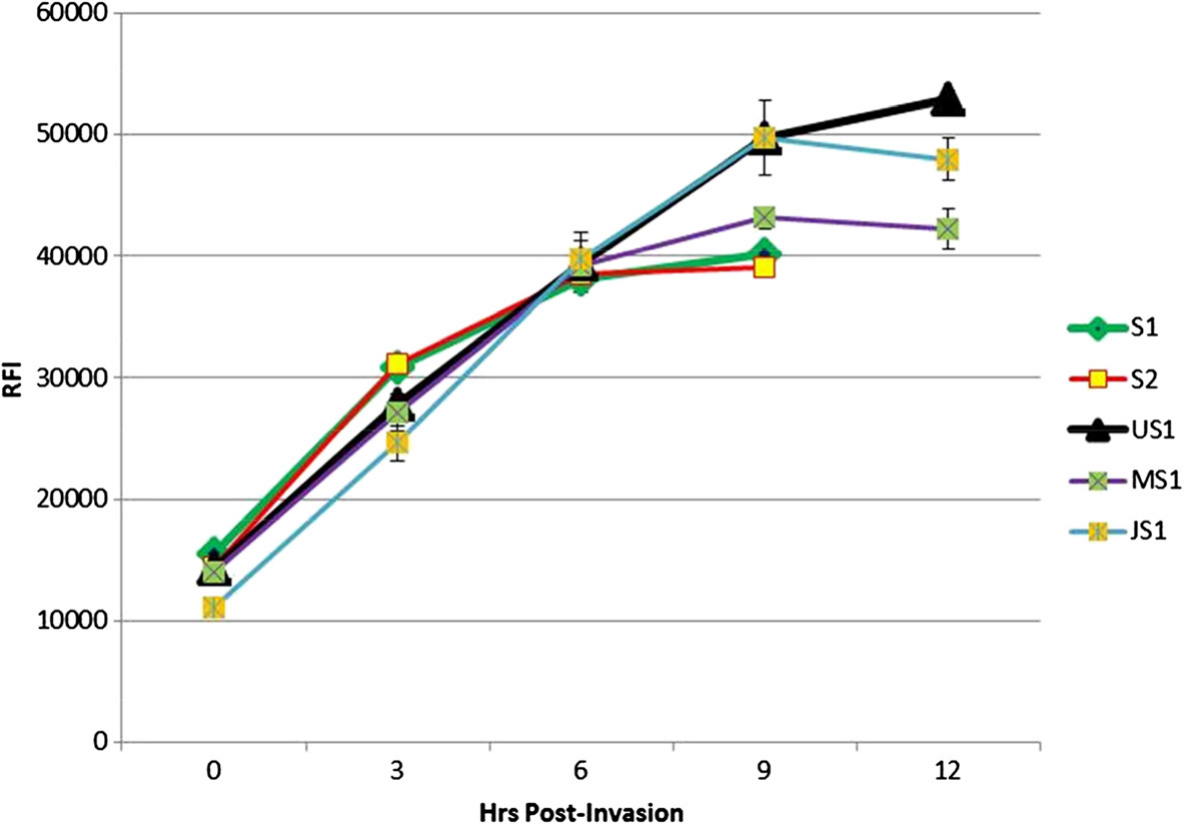
**Synchrony comparison of culture synchronization methods.** For JS1, parasites were synchronized as outlined in Miao *et al.*[10]. MS1 parasites were treated similarly to JS1 except sorbitol treatment was omitted. S1 and S2 preparations represent two fractions of the Percoll synchronization method described here. For the unsynchronized culture (US1), schizonts were isolated from a 20% over 60% Percoll cushion. Following synchronization, schizonts were cultured as previously described in 1% haematocrit, and aliquots were taken every 3-hr (starting at 0-hr up to 12-hr). Samples were frozen, thawed, and subjected to SyBr Green I assay. RFI units are listed on the left of the graph. All data points indicate average and standard deviations from triplicate samples.

Figure 3 shows the comparison for the degrees of synchrony between Percoll synchronized samples (S1: 0- to 3-hr and S2: 3- to 6-hr) to that obtained from: (1) unsynchronized schizonts isolated from a one-step procedure of 20% over 60% Percoll cushion (US1); (2) a culture synchronized by the Percoll/sorbitol treatment method (JS1); and (3) a mock synchronized control culture treated similar to JS1 above except without sorbitol treatment (MS1). The results show that the unsynchronized parasites isolated on 60% Percoll (US1) continued its RFI increase beyond 9-hr. In contrast, the first two preparations (S1 and S2) were synchronized within the range of 4- to 6-hr. The Percoll/sorbitol synchronized culture produced a 9-hr synchrony period (JS1), while the mock culture (MS1) closely mimicked preparations S1 and S2. These results indicate that sorbitol treatment may not actually improve culture synchrony.

## Conclusion

In this study, an alternative method for the synchronization of *Plasmodium falciparum* cultures was developed, demonstrating that Percoll density centrifugation alone is sufficient to synchronize the parasite cultures. Furthermore, a procedure that tests the degree of culture synchrony was established; this procedure is objective, easy to perform, and can validate the degree of synchrony regardless of synchronization method employed. These two techniques are anticipated to be useful for studying late-stage developmental processes associated with *Plasmodium falciparum* egress.

## Competing interests

The authors declare that they have no competing interests.

## Authors’ contributions

RC designed and performed the experiments, and wrote the manuscript. JM performed Percoll/sorbitol synchronizations and provided invaluable advice. LC and CG reviewed and approved the final manuscript. All authors read and approved the final manuscript.

## Supplementary Material

Additional file 1 **4- to −6 hr synchronization protocol.** A detailed description of the synchronization protocol developed and used for this paper. Click here for file

Additional file 2 **Confirmation of culture synchrony.** A detailed description of the synchrony validation method used for this paper. Click here for file

Additional file 3 **SyBr Green analysis of lifecycle using a culture synchronized by the alternative Percoll method used in this paper.** Additional file 3 is similar to Figure 1 except the culture was synchronized using the Percoll synchronization method and assayed for Total DNA *versus* Live Cell DNA rather than cells with or without RNase A. Synchronized 3D7 schizonts were isolated and allowed to infect uRBCs. Culture was run over a 40% and 60% Percoll cushion to recover only schizonts. Schizonts were washed with RPMI, pelleted, then re-suspended in complete media containing 1% RBCs and returned to culture. At the indicated time points following egress, aliquots were either harvested with (live cell DNA) or without (total DNA) a 40% Percoll centrifugation. Collected aliquots were frozen and after thawing, a SyBr Green assay was performed. Click here for file

Additional file 4 **Conceptual rationale behind the alternative synchronization method.** Theoretical outline depicting the progress of a synchronized *Plasmodium* culture. The illustration depicts a 3-hr period for each set of Percoll captured RS. The long arrows point from the culture status (ie. bell-shaped graph) towards time (i e, 0-hr, 3-hr, 6-hr, etc.), denoting the development of the culture. As time progresses, the synchronized culture will develop from TS to SS then RS. The beginning of lysis marks time 0-hr for this illustration. At time equals 3-hr the fraction of SS that develop into RS is noted as S1. This S1 fraction of rings is collected by 70% Percoll centrifugation. By 6-hr the fraction of SS that develops into RS is noted as S2 and is subsequently harvested by 70% Percoll centrifugation. The last fraction of SS to develop into RS is S3 and it occurs at 9-hr. Each fraction (S1 through S3) has its own 0-hr start time which is 3-hr after its predecessor. Each fraction will begin the egress/invasion process 48-hr from the time it is harvested. Each fraction is 4- to 6-hr synchronized with fraction S2 having the highest parasitaemia (Additional file 6). Click here for file

Additional file 5 **Giemsa stains of parasite lifecycle.** Pictures are Giemsa stains representing aliquots of “total DNA” taken at specified time intervals (Additional file 1). Click here for file

Additional file 6 **Parasite concentration from 3 hr-synchronized cultures.** Cultures were synchronized as detailed in the Methods section. Aliquots of each synchronized 3-hr period (S1, S2, and S3) were taken to determine which preparation possessed the highest parasite concentration as measured by the SyBr Green assay. S1 represent the 0- to −3 hr aliquot. S2 and S3 represent the 3- to 6-hr and 6- to 9-hr aliquots respectively. Results are averages from two independent cultures each ran in triplicate. Click here for file

Additional file 7 **Isolated schizonts from step 5.** Giemsa stain of schizonts isolated from step 5 of the Percoll synchronization protocol (Additional file 4). Click here for file

Additional file 8 **Isolated schizonts from step 8.** Giemsa stain of schizonts isolated from step 8 of the Percoll synchronization protocol (Additional file 4). Click here for file

Additional file 9 **Isolated rings from step 10.** Giemsa stain of rings isolated from step 10 of the Percoll synchronization protocol (Additional file 4). Click here for file

Additional file 10 **3-hr rings.** Giemsa stain of 3-hr rings taken from the synchrony confirmation protocol (Additional file 5). Click here for file

Additional file 11 **6-hr rings.** Giemsa stain of 6-hr rings taken from the synchrony confirmation protocol (Additional file 5). Click here for file

Additional file 12 **9-hr rings.** Giemsa stain of 9-hr rings taken from the synchrony confirmation protocol (Additional file 5). Click here for file
