# Supplementary material for: An alternative protocol for Plasmodium falciparum culture synchronization and a new method for synchrony confirmation
Source: Malar J. 2013 Nov 1;12:386. doi: 10.1186/1475-2875-12-386 (PMC3819685; doi:10.1186/1475-2875-12-386)
Supplement: Additional file 1 — 4- to −6 hr synchronization protocol. A detailed description of the synchronization protocol developed and used for this paper. [file 1475-2875-12-386-S1.pdf]

4hr-6hr Pf synchronization protocol

Semi-synchronization steps (T75 flask)

To grow culture to high parasitemia (10% or higher) and partially synchronous, proceed as follows:

1. Change media by aspirating at the 24hr-30hr stage (early to mid TS).
2. Isolate schizonts using 20%/60% percoll step-gradient (2x 15ml tubes).

NOTE: Do not wash schizonts and minimize recovered percoll volumes to less than 2mls.

3. Resuspend schizonts in 50mls complete media containing 1% RBCs.
4. Repeat as necessary until a semi-synchronized high parasitemia is obtained (10% or higher).

Synchronization steps

5. Isolate schizonts (step 2), and resuspend them in 25mls complete media containing 2% RBCs.
6. Incubate for 3hrs (upright standing position), then run over a 20%/70% percoll step-gradient.
7. Save the 70% layer (schizonts) and the pellet (rings).
8. Schizonts: Repeat steps 5-7 for a total of three times (never wash or pellet schizonts).
9. Rings: wash rings with ~12mls-14mls RPMI and pellet.

Optimization steps

10. Resuspend rings in 25mls complete media (pre-warmed).

NOTE: Document start-time and packed cell volume (PCV). Make smears and take 0.5ml aliquot. Adjust aliquot to 1% hematocrit, pellet, and freeze. Determine the RFI value of all 3 samples using the SyBr Green assay. All samples are 6hr synchronized but at different parasitemia.

11. Incubate for 7-9hrs, then run over a 40%/70% percoll step-gradient and save pellets.
12. Wash pellet with 12mls-14mls RPMI, pellet and resuspend in 50mls complete media.
13. Incubate 20hrs-24hrs, aspirate media, then replenish with 50mls complete media.

NOTE: Harvest 46hrs-48hrs from step 10 (documented start time) as follows:

14. Aspirate media leaving 18-20mls in the flask, then run over a 20%/60% percoll step-gradient.
15. Save schizonts, which are now 4-6hr synchronized. Test synchrony and/or use for experiments.

Additional NOTES:

80% Percoll may be used in place of 70% Percoll for slightly higher RS purity

All spins are 2000 x G for 6min @ RT in a swinging bucket rotor.

All percoll volumes during extraction are <2mls.

All incubations are in CO_2 incubator @ 37°C and media is warmed to 37°C before use.

Never wash schizonts and keep recovered percoll volumes to a minimum (i.e. less than 5% V/V).
