# Supplementary material for: An alternative protocol for Plasmodium falciparum culture synchronization and a new method for synchrony confirmation
Source: Malar J. 2013 Nov 1;12:386. doi: 10.1186/1475-2875-12-386 (PMC3819685; doi:10.1186/1475-2875-12-386)
Supplement: Additional file 2 — Confirmation of culture synchrony. A detailed description of the synchrony validation method used for this paper. [file 1475-2875-12-386-S2.pdf]

Confirmation of culture synchrony

1. Isolate SS over a 20%/60% percoll step-gradient, minimizing percoll recovery.
2. Resuspend isolate in at least 20 volumes of pre-warmed complete media containing 1% RBCs, and incubate at 37°C in CO_2 incubator.
3. Remove 1ml aliquots every 2hrs-3hrs starting from 0hrs and ending @ 9hrs-12hrs.
4. Run 0.5ml aliquots over 70% percoll (0.5mls) in 1.6ml eppendorf tubes and spin @ 4000 x G for 6min at rt.
5. Wash RS pellet with 1.5mls of RPMI and freeze for later analysis.
6. When ready to process, thaw samples by resuspending in SyBr Green resuspension buffer.
7. Process via the SyBr Green detection method and analyze results.
