# Supplementary figures and images for: An alternative protocol for Plasmodium falciparum culture synchronization and a new method for synchrony confirmation
Source: Malar J. 2013 Nov 1;12:386. doi: 10.1186/1475-2875-12-386 (PMC3819685; doi:10.1186/1475-2875-12-386)

Begin egress/invasion

TS → SS  
Transition

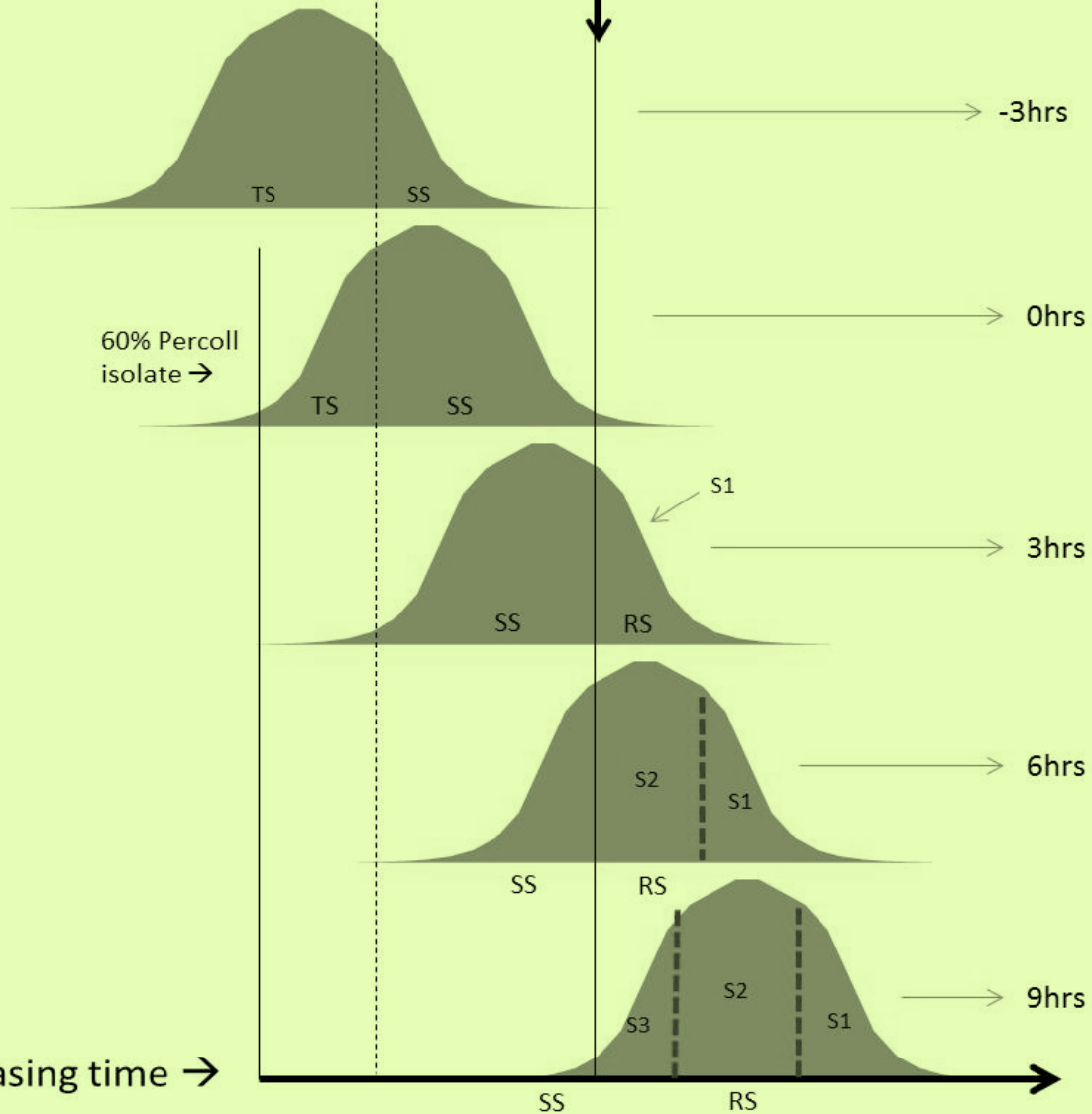

Supplement: Additional file 3 — SyBr Green analysis of lifecycle using a culture synchronized by the alternative Percoll method used in this paper. Additional file 3 is similar to Figure 1 except the culture was synchronized using the Percoll synchronization method and assayed for Total DNA versus Live Cell DNA rather than cells with or without RNase A. Synchronized 3D7 schizonts were isolated and allowed to infect uRBCs. Culture was run over a 40% and 60% Percoll cushion to recover only schizonts. Schizonts were washed with RPMI, pelleted, then re-suspended in complete media containing 1% RBCs and returned to culture. At the indicated time points following egress, aliquots were either harvested with (live cell DNA) or without (total DNA) a 40% Percoll centrifugation. Collected aliquots were frozen and after thawing, a SyBr Green assay was performed. [file 1475-2875-12-386-S3.pdf]

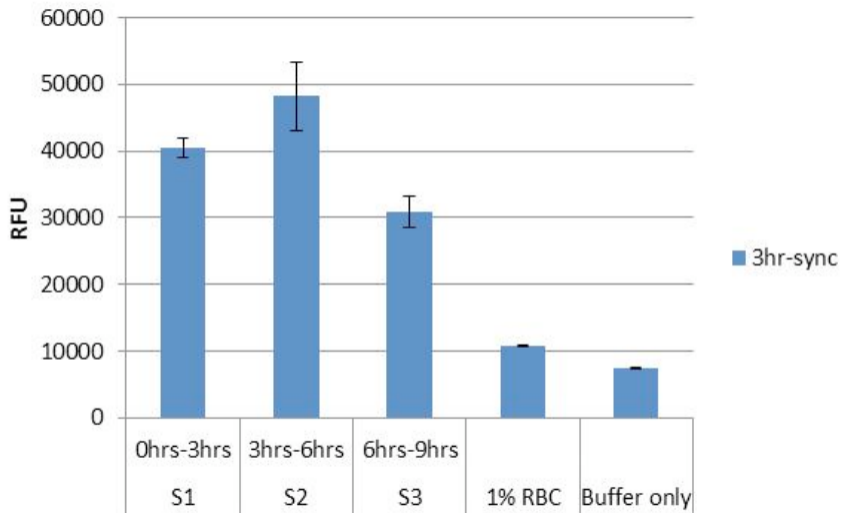

Supplement: Additional file 4 — Conceptual rationale behind the alternative synchronization method. Theoretical outline depicting the progress of a synchronized Plasmodium culture. The illustration depicts a 3-hr period for each set of Percoll captured RS. The long arrows point from the culture status (ie. bell-shaped graph) towards time (i e, 0-hr, 3-hr, 6-hr, etc.), denoting the development of the culture. As time progresses, the synchronized culture will develop from TS to SS then RS. The beginning of lysis marks time 0-hr for this illustration. At time equals 3-hr the fraction of SS that develop into RS is noted as S1. This S1 fraction of rings is collected by 70% Percoll centrifugation. By 6-hr the fraction of SS that develops into RS is noted as S2 and is subsequently harvested by 70% Percoll centrifugation. The last fraction of SS to develop into RS is S3 and it occurs at 9-hr. Each fraction (S1 through S3) has its own 0-hr start time which is 3-hr after its predecessor. Each fraction will begin the egress/invasion process 48-hr from the time it is harvested. Each fraction is 4- to 6-hr synchronized with fraction S2 having the highest parasitaemia (Additional file 6). [file 1475-2875-12-386-S4.pdf]

6hr

12hr

18hr

24hr

30hr

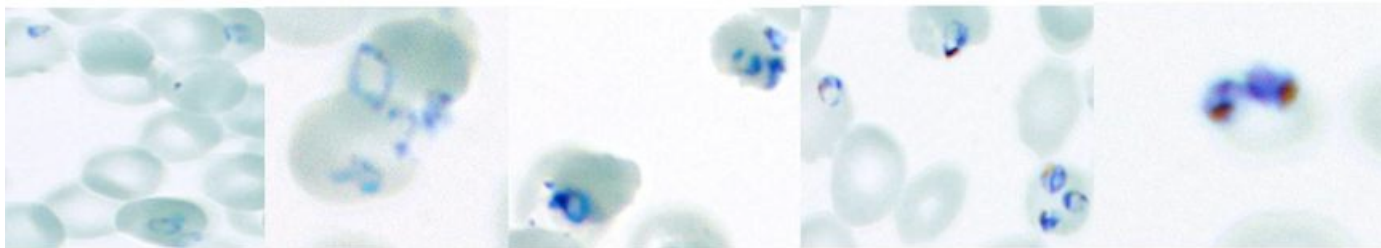

36hr

42hr

48hr/0hr

54hr/6hr

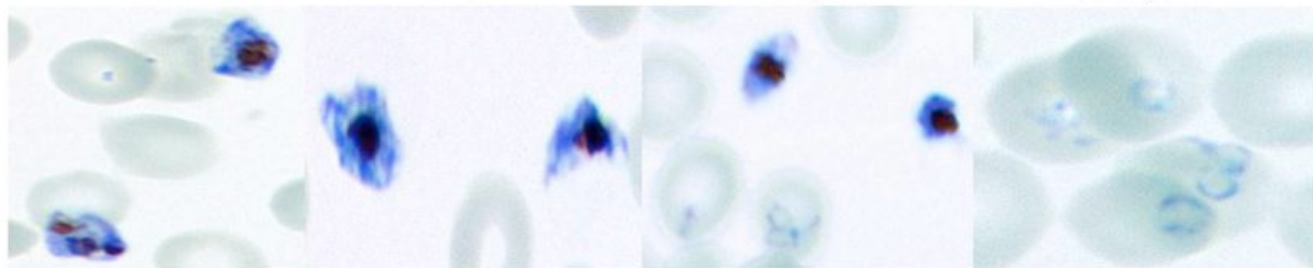

60hr/12hr

66hr/18hr

72hr/24hr

78hr/30hr

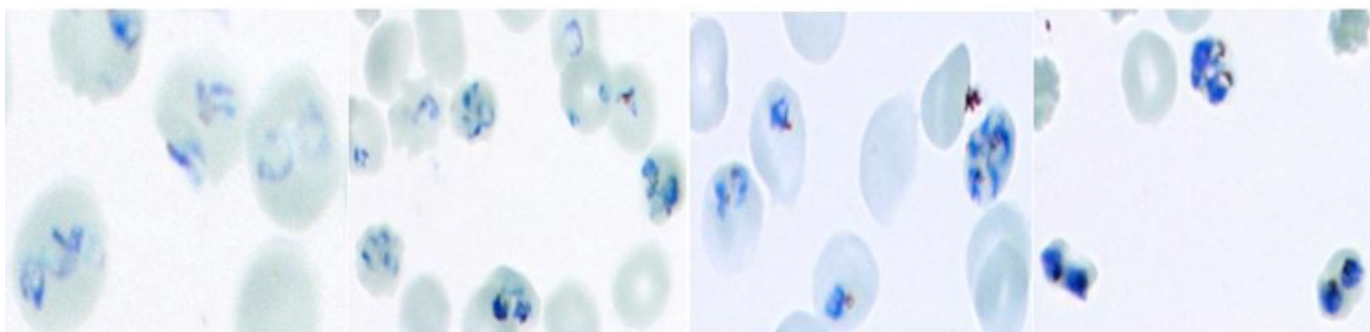

84hr/36hr

90hr/42hrs

96hr/48hrs

102hr/6hrs

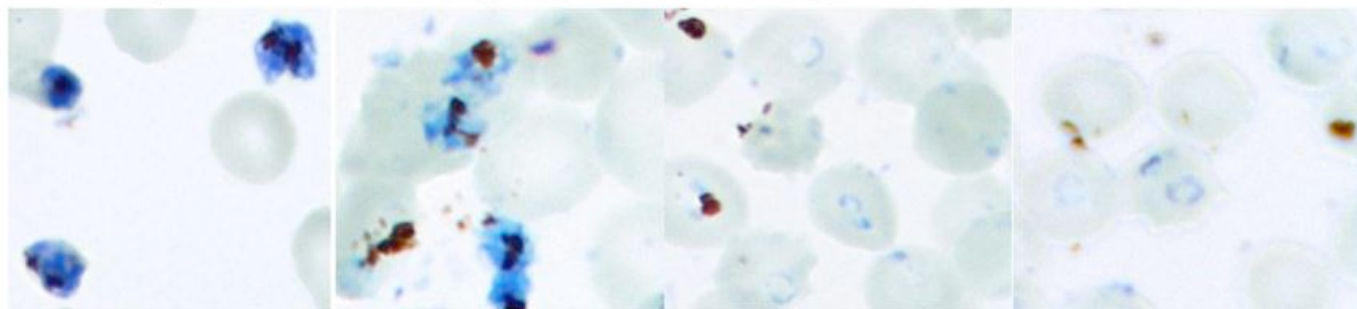

Supplement: Additional file 5 — Giemsa stains of parasite lifecycle. Pictures are Giemsa stains representing aliquots of “total DNA” taken at specified time intervals (Additional file 1). [file 1475-2875-12-386-S5.pdf]

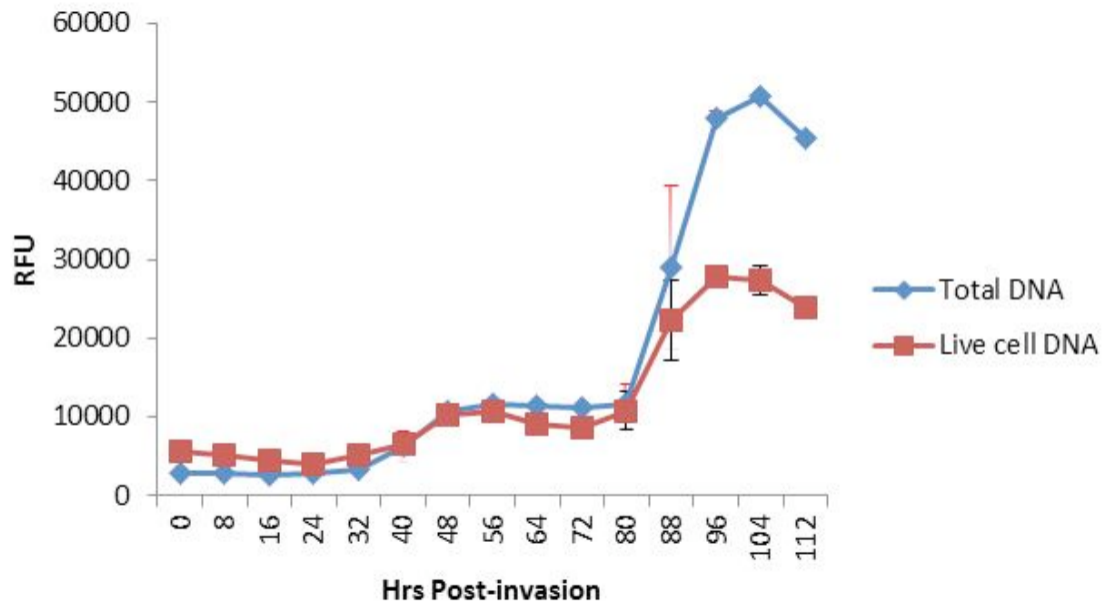

Supplement: Additional file 6 — Parasite concentration from 3 hr-synchronized cultures. Cultures were synchronized as detailed in the Methods section. Aliquots of each synchronized 3-hr period (S1, S2, and S3) were taken to determine which preparation possessed the highest parasite concentration as measured by the SyBr Green assay. S1 represent the 0- to −3 hr aliquot. S2 and S3 represent the 3- to 6-hr and 6- to 9-hr aliquots respectively. Results are averages from two independent cultures each ran in triplicate. [file 1475-2875-12-386-S6.pdf]

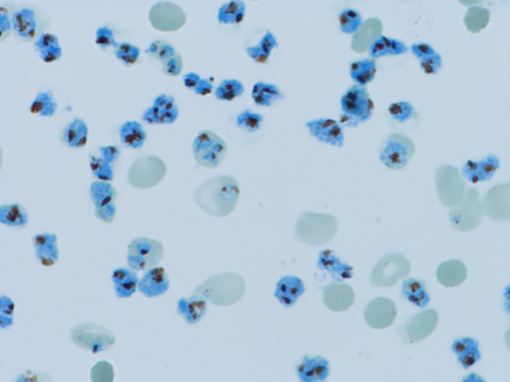

Supplement: Additional file 7 — Isolated schizonts from step 5. Giemsa stain of schizonts isolated from step 5 of the Percoll synchronization protocol (Additional file 4). [file 1475-2875-12-386-S7.pdf]

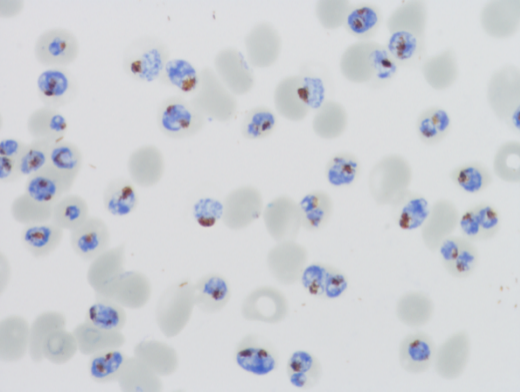

Supplement: Additional file 8 — Isolated schizonts from step 8. Giemsa stain of schizonts isolated from step 8 of the Percoll synchronization protocol (Additional file 4). [file 1475-2875-12-386-S8.pdf]

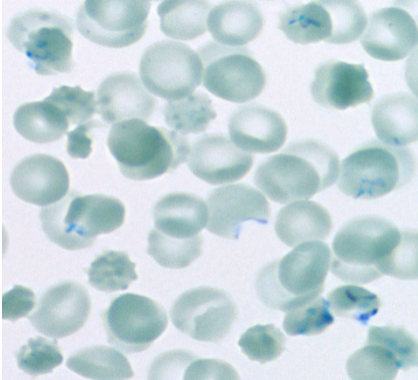

Supplement: Additional file 9 — Isolated rings from step 10. Giemsa stain of rings isolated from step 10 of the Percoll synchronization protocol (Additional file 4). [file 1475-2875-12-386-S9.pdf]

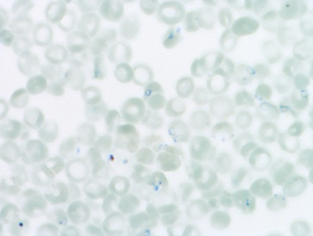

Supplement: Additional file 10 — 3-hr rings. Giemsa stain of 3-hr rings taken from the synchrony confirmation protocol (Additional file 5). [file 1475-2875-12-386-S10.pdf]

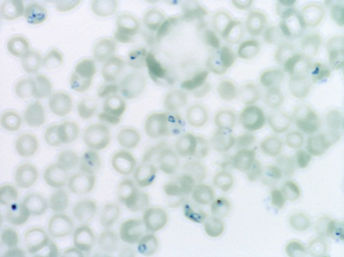

Supplement: Additional file 11 — 6-hr rings. Giemsa stain of 6-hr rings taken from the synchrony confirmation protocol (Additional file 5). [file 1475-2875-12-386-S11.pdf]

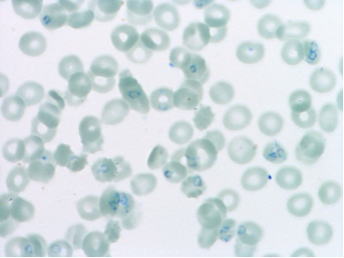

Supplement: Additional file 12 — 9-hr rings. Giemsa stain of 9-hr rings taken from the synchrony confirmation protocol (Additional file 5). [file 1475-2875-12-386-S12.pdf]
